# Effect of Zinc, Magnesium, and Manganese Phosphate Coatings on the Corrosion Behaviour of Steel

**DOI:** 10.3390/ma18133126

**Published:** 2025-07-01

**Authors:** Alin-Marian Cazac, Lucian-Ionel Cioca, Petru Lazar, Gheorghe Badarau, Nicanor Cimpoesu, Diana-Petronela Burduhos-Nergis, Pompilica Iagaru, Ramona Cimpoesu, Anca Cazac, Costica Bejinariu, Adriana Milea (Pârvu)

**Affiliations:** 1 Faculty of Materials Science and Engineering, Gheorghe Asachi Technical University of Iasi, 67 Dimitrie Mangeron str., 700050 Iasi, Romania; alin-marian.cazac@academic.tuiasi.ro (A.-M.C.); lazar.petru@gmail.com (P.L.); gheorghe.badarau@academic.tuiasi.ro (G.B.); nicanor.cimpoesu@academic.tuiasi.ro (N.C.); diana-petronela.burduhos-nergis@academic.tuiasi.ro (D.-P.B.-N.); anca.cazac@student.tuiasi.ro (A.C.); costica.bejinariu@academic.tuiasi.ro (C.B.); 2 Faculty of Engineering, Lucian Blaga University of Sibiu, 10 Victoriei Blvd., 550024 Sibiu, Romania; adriana.milea@ulbsibiu.ro; 3 Academy of Romanian Scientists, Ilfov 3, 050044 Bucharest, Romania; 4 Faculty of Agricultural Sciences, Food Industry and Environmental Protection, Lucian Blaga University of Sibiu, 7-9 dr. Ion Ratiu Street, 550012 Sibiu, Romania; pompilica.iagaru@ulbsibiu.ro

**Keywords:** corrosion resistance, coatings, microstructure, steel, reinforced concrete

## Abstract

This study provides a systematic comparison of three types of phosphate coatings, applied by identical immersion phosphating processes and tested under two different environmental conditions representative of real industrial scenarios. The focus of this study is the investigation of the corrosion behaviour of zinc, magnesium, and manganese phosphate coatings on reinforcing steel in two different corrosion environments: river water and seawater. The phosphate coatings were obtained via the immersion phosphating technique. Various techniques, including scanning electron microscopy (SEM), energy dispersive X-ray spectroscopy (EDX), potentiodynamic polarization curve (PDP) testing, and electrochemical impedance spectroscopy (EIS), were used to evaluate the morphology and corrosion resistance of the coatings. The overall corrosion protection performance of the coatings followed the order of Zn phosphate > Mn phosphate > Mg phosphate. The results indicate that the samples with the Zn-phosphated coating showing the highest improvement. This coating showed no major morphological changes and achieved significantly reduced corrosion rates—0.258 µm/year in river water and 3.060 µm/year in seawater—compared to the typical corrosion rate of uncoated steel, which is about 45 µm/year. These findings emphasize the effectiveness of Zn phosphate coatings in mitigating corrosion in both river water and marine conditions.

## 1. Introduction

Reinforced concrete is a structural material widely used in civil engineering projects such as buildings, dams, bridges, etc. [1]. These structures are complex to build, very expensive, and have a limited-service life. Reinforced concrete structures can be affected by physical deterioration (cracking, frost, fire), chemical deterioration (sulphate attack, acid attack, seawater, etc.) and reinforcement corrosion [2,3].

Various methods are commonly used to minimize rebar corrosion, such as cathodic protection, recalcination, and the external application of coatings to the concrete surface or rebars [4]. Another alternative is using inhibitors, which can be cost effective and easy to apply. They can be used in concrete reinforcement by adding the inhibitor agent along with water during concrete preparation or by applying it to the outer surface of the hardened concrete. Reviews of the most common types of inhibitors used in concrete repair and the different inhibition mechanisms are available in the literature [5]. Most commonly, mixed inhibitors are based on nitrite ions [6,7]. However, nitrites should be used with caution, as they may contaminate the surrounding soil or water.

Other ions have been studied to inhibit steel corrosion, i.e., chromium, phosphorus, tungsten, and molybdenum ions [8]. When reviewing the studies, it can be observed that phosphates exhibit a number of interesting advantages such as low cost and low toxicity. To evaluate the effectiveness of phosphate ions as inhibitors for reinforced concrete, some authors have simulated porous solutions [9]. In contrast, few articles have reported the effectiveness of phosphate ions in mortars or grouts under long-term exposure [10]. The interaction of phosphate ions with grouts is complex. Some authors claim that phosphates can change the mechanical properties of concrete or alter hardening rates by decomposing and precipitating as calcium phosphate, reducing the inhibitor’s effectiveness [11,12]. However, other authors have reported that this inhibitor does not interfere with the concrete and has been shown to be effective in mortars [10,11]. In addition, the optimal phosphate/chloride ratio in mortars is controversial, and the inhibition mechanism is not clear. Some authors propose a dual effect, where calcium phosphate could block pores to prevent diffusion, while phosphate could block cathodic or anodic sites [10,13,14].

Worldwide, steel reinforcement corrosion is a major concern, as premature failure of concrete structures leads to safety issues, along with huge repair and replacement costs [15]. In an alkaline environment such as concrete (pH > 10), steel is normally passivated. However, there are two main reasons for the passivated layer to decompose. On one hand, the penetration of aggressive ions (e.g., chloride ions) can cause localised failure of the passivation layer, leading to localised corrosion. Moreover, carbonation causes a decrease in pH, and once the value falls below 10, the protective passivation layer becomes unstable. This loss of passivation increases the susceptibility of the steel surface to generalized corrosion [15].

Common corrosion inhibitors include nitrites (calcium nitrite and sodium nitrite) and various organic inhibitors such as amino alcohols and amines (in some cases, mixtures are also used); sodium monofluorophosphate (MFP) is also regularly used as a surface-applied inhibitor [16,17,18,19].

Phosphating is the process of depositing a layer of soluble phosphate compounds on the surface of a metal by conversion. Although phosphate coatings have been studied since the beginning of the 19th century, they are not only still being studied, but they have also become a major focus, given their numerous applications. The advantages of this type of coating are well known, such as the low cost of the deposition process, improved corrosion resistance properties, improved wear resistance and the possible adhesion of subsequent deposited coatings such as paint. Therefore, studies are being conducted to continuously improve the properties of the phosphate coating by modifying the parameters of the phosphating process and by modifying/replacing the substances used in the phosphating solutions with “environmentally friendly” types. In addition, these benefits have led several researchers to investigate phosphate coatings for applications in civil engineering and medicine (biomaterial coatings) [20].

Zn phosphating is one of the most widely used surface treatments and is typically performed in aqueous acid phosphate solutions containing zinc and phosphoric acid ions, with nitrate ions as an accelerator to promote oxidation and dissolution of the metal surface [21]. The phosphate coating is known to develop through the nucleation, growth, and coalescence of zinc phosphate crystals. The corrosion resistance of the phosphate coating is related to the size and density of the pores in the coating [22]; i.e., the pores provide a pathway for corrosion attack [23].

Focus areas for improving phosphating processes are enhancing the protective and other functional properties of coatings, reducing solution concentration, temperature, and processing time, incorporating adjustments, standardising phosphating compositions, and reducing the environmental risk. Given its use in civil engineering, reinforcing steel must be as durable as possible at all levels. The reinforcement is the central part of a structure that must last a very long time. The immersion phosphating process is a relatively simple and inexpensive method of obtaining phosphate layers that can improve the properties of reinforcing steel. The main property that benefits is corrosion resistance, which extends the application areas of this steel.

A new aspect of this investigation is the systematic comparison of three types of phosphate coatings applied by identical immersion phosphating processes under two different environmental conditions relevant to industrial applications. This study also contributes to a new comparative perspective on the performance of phosphate coatings in both river and marine environments, supporting the informed selection of corrosion attenuation strategies for steel-reinforced concrete structures. This study investigates the effect of phosphate coatings on the corrosion behaviour of reinforcing steel using three different phosphating solutions. The substrate material used in the experiments was a reinforcing steel. The corrosion experiments were performed in two distinct aqueous environments—marine (seawater) and fluvial (river water)—with the resulting data and analysis detailed in this study.

## 2. Materials and Methods

### 2.1. Test Material

The experimental tests were performed on reinforcing steel samples used for concrete structure reinforcement, the characteristics having been presented in a previous article [1]. Concrete steel is a carbon steel that has a smooth profile and is hot rolled. Reinforcing steel bars were used, and samples measuring 10 mm in diameter and 3 mm in height were cut from each bar. The chemical composition of the steel, obtained by spark spectroscopy and provided by the supplier, is presented in Table 1.

The steel specimens were prepared and treated with zinc, magnesium, and manganese phosphate solutions by immersion phosphating to produce protective coatings for comparative evaluation of their corrosion performance under realistic environmental conditions.

### 2.2. Phosphating Process

The process steps for phosphating involved degreasing, pickling, phosphating, and drying. The immersion phosphating method was employed because the metal surface was uniformly covered, and the sample size allowed this. The three phosphating solutions used were as follows: solution I/Mg, solution II/Zn, and solution III/Mn, presented in Table 2 [1].

The pH value of these solutions was determined using a RADELKIS OP-264/1 RADELKIS OP-264/1 pH meter (Radelkis, Budapest, Hungary) in the laboratory of the Faculty of Chemical Engineering and Environmental Protection “Cristofor Simionescu”, Technical University “Gheorghe Asachi”, Iasi.

### 2.3. Electrochemical Tests

The equipment used was an OrigaFlex OGF+01A potentiostat (OrigaLys ElectroChem SAS, Rillieux-la-Pape, France) with a three-electrode electrochemical cell containing a platinum auxiliary electrode, a calomel reference electrode, and a working electrode. The samples measured 7 mm in diameter and 3 mm in thickness (exposed surface area of 0.384 cm^2^). The following electrochemical methods were employed to analyse the corrosion behaviour: open circuit potential (OCP), linear voltammetry (LV), and cyclic voltammetry (CV). The potentiodynamic curves were recorded at a scanning rate of 0.5 mV/s and a potential range of −350 to +400 mV (SCE). For cyclic voltammetry tests, the initial potential was −400 mV (SCE), and the final potential was +500 mV (SCE), with a scan rate of 10 mV/s (SCE). For electrochemical impedance spectroscopy measurements, the working potential = the open circuit potential, with a frequency range from 10^5^ to 0.1 Hz and a current amplitude 10 mV (SCE). All the test were conducted at room temperature (22 °C). All measurements (potentiodynamic polarization, EIS) were repeated three times for each sample type to ensure reproducibility and statistical reliability, and average values were reported.

Two types of water (river water and seawater) were chosen as the electrolyte. The river water was taken from the Bahlui River, a local river, and the seawater was taken from the Aegean Sea. The curves obtained were recorded after the samples had been immersed in the solution for 60 min. The measurements were registered via OrigaMaster 5 (v.2.5.0.5) data acquisition and processing software. To evaluate the corrosion behaviour, electrochemical tests were conducted in the two electrolyte solutions, i.e., the uncoated reinforcing steel specimens and those treated with the three distinct phosphating coatings.

## 3. Results

### 3.1. Corrosion Behaviour Evaluation

**Figure 1:**
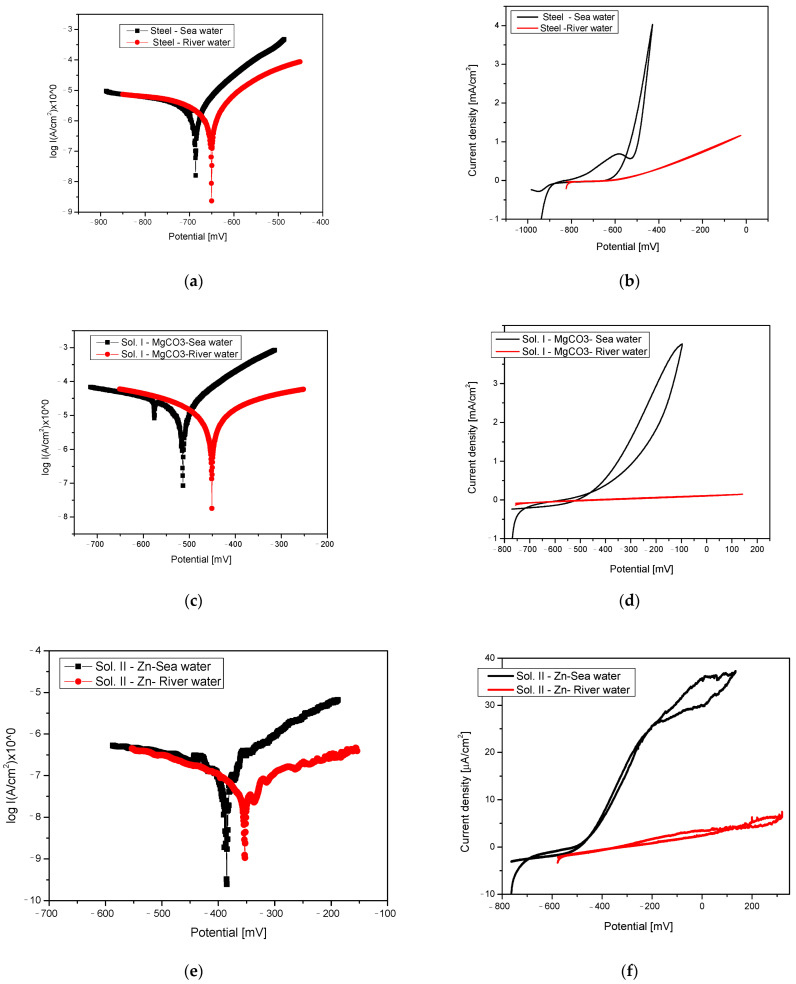

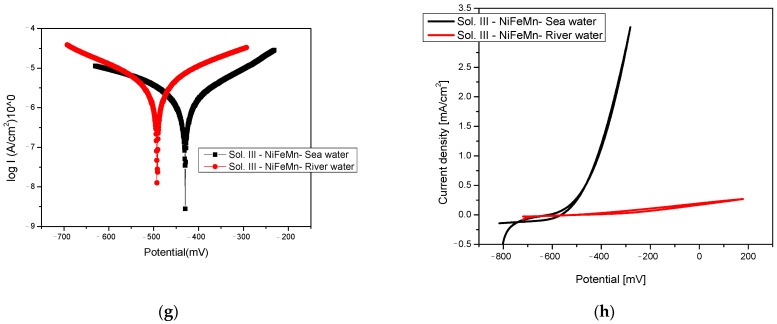
Polarisation curves, Tafel diagram, and cyclic diagram obtained in river water and seawater for (**a**,**b**) reinforcing steel; (**c**,**d**) I/Mg solution; (**e**,**f**) II/Zn solution; (**g**,**h**) III/Mn solution.

Figure 1a,b shows the polarisation diagrams for reinforcing steel obtained in both seawater and river water electrolyte solutions. For both cases, it can be observed that the anodic reaction, i.e., the oxidation of Fe—reaction (1), is predominant compared to the cathodic type—reaction (2). In these aqueous media, corrosion occurs in the presence of oxygen. The corrosion of Fe in aqueous media starts with the anodic reaction:

Fe → Fe^+2^ + 2e^−^, (1)

and the cathodic reaction:

H_2_O + 1/2O_2_ + 2e^−^ → 2OH^−^, (2)

The initial layer formed in direct contact with the Fe surface consists primarily of iron and oxygen. This interfacial layer is generated as a result of the reaction between Fe^2^⁺ ions and hydroxide ions (OH^−^), as described by reactions (3) and (4):

Fe^+2^ + 2OH^−^ → Fe(OH)_2_, (3)

Further oxidation of iron into Fe^+3^ occurs according to the following reaction:

4Fe(OH)_2_ + 2H_2_O + O_2_ → 4 Fe(OH)_3_ (4)

Reactions (1) to (4) lead to the formation of hydroxides as a result of corrosion in aqueous solutions.

The cyclic voltammetry curves presented in Figure 1b reveal the distinct electrochemical behaviour of the reinforcing steel samples in the two electrolyte solutions. The diagram of the reaction in river water shows a generalised corrosion process over the entire surface of the alloy, without any passivation phenomena. In this case, the current density remains constant and is much lower than that in seawater. The cyclic diagram recorded in seawater shows a pre-corrosive character at points where the current density value increases significantly when metal oxidation processes occur. At higher potentials, oxygen evolution can occur in conjunction with metal oxidation [17]. This can be attributed to the increased concentration of Cl^−^ ions in seawater [13]. Chloride ions can react with Fe ions in an autocatalytic reaction, resulting in the formation of pitting corrosion [18]:

Fe^+2^ + 2Cl^−^ → FeCl_2_ + 2H_2_O → Fe(OH)_2_ + 2HCl (5)

Figure 1c,d present the potentiodynamic polarization curves corresponding to the samples treated with phosphating solution I. In this case the phosphating layer is enriched with Mg ions. A comparable electrochemical response is observed in the linear polarization measurements of the samples in both solutions, characterized by the predominance of the anodic process [24,25]. The corrosion of Mg in an aqueous solution occurs in three steps, involving the following reactions:

First stage—anode reaction:

Mg → Mg^2+^ + 2e^−^ (6)

Second stage—cathodic reaction:

2H_2_O + 2e^−^ → H_2_ + 2OH^−^ (7)

and the corrosion product formation:

Mg^2+^ + 2OH^−^ → Mg(OH)_2_ (8)

Mg + 2H_2_O → Mg(OH)_2_ + H_2_ (9)

Cyclic polarization (Figure 1d) shows a similar behaviour to that of the reinforcing steel sample, presumably caused by the high reactivity of the Mg layer, which led to its breakthrough and the electrolyte reaching the substrate.

Figure 1e,f display the linear and cyclic polarization curves for samples treated with phosphating solution II, tested in river water and seawater, respectively. No significant variation is observed between the anodic and cathodic Tafel slopes, indicating that the oxidation process associated with metal corrosion proceeds at a relatively slow rate [21]. This phenomenon is attributed to the presence of zinc within the phosphate layer, which, upon exposure to the aqueous environment, forms zinc oxide (ZnO), a passivating oxide film [22].

When Zn is corroded by an aqueous medium, the following redox reactions occur:

Zn → Zn^+2^ + 2e^−^ (10)

2H_2_O + O_2_ + 4e^−^ → 4OH^−^ (11)

and the zinc oxide formation reactions are as follows:

Zn^+2^ + 2OH^−^ → Zn(OH)_2_ (12)

2Zn^+2^ + 2OH^−^ → 2ZnO + H_2_ (13)

The cyclic polarization shown in Figure 1f confirms the formation of the formed oxide layer. The figure outlines a passive region followed by the initiation of pits, where a current hysteresis loop is then formed that is typical for pit attack. The current density of the backward scan was higher than that of the forward scan, indicating the breakdown of the ZnO layer. After the pitting potential, around −380 mV (SCE) in this case, the metal starts to corrode.

Figure 1g,h present the Tafel and cyclic polarization curves, respectively, for samples treated with phosphating solution III. In this case as well, no significant difference was observed between the anodic and cathodic slopes, with the corrosion of the layer occurring at a much slower rate than for reinforcing steel. The cyclic polarisation curves show a difference in the corrosion behaviour in both environments (Figure 1h), where the samples showed a generalised corrosion pattern. The graph indicates a uniform corrosion process occurring across the entire surface. The return curve (cathodic branch of the polarisation curve) exactly overlaps with the cathodic branch, indicating that no other processes (passivation, transpassivation) have occurred in this area. This phenomenon may be explained by the complex composition of the Ni, Fe, and Mn phosphate layer.

Table 3 shows the corrosion process parameters derived from Tafel diagrams for all phosphated samples and for reinforcing steel.

The best corrosion resistance, along with the lowest corrosion rate, was recorded for the samples phosphated with Zn solution in both electrolyte media, i.e., 0.258 µm/year for river water and 3.060 µm/year for seawater. The recorded values were significantly lower than the corrosion rate of unprotected steel, which is approximately 45 µm/year.

Regarding the corrosion potential E (I = 0), which expresses the corrosion tendency of the alloy immersed in an electrolytic medium, the samples phosphated with II/Zn solution show the best behaviour. The lowest corrosion potential value recorded was −353 mV (SCE) for the II/Zn solution in river water, compared with −650.6 mV (SCE) for reinforcing steel in the same electrolyte. The corrosion current values determined as the corrosion potential of the alloy using the linear polarisation curve actually represent the corrosion current that occurs at the metal/corrosive interface when the metal is immersed in the solution. It is inversely proportional to the value of the polarisation resistivity [23]. The lowest corrosion current was recorded on samples phosphated with the II/Zn solution. The highest corrosion current with the strongest oxidation reaction was recorded for the I/Mg solution due to the high reactivity of Mg.

The of electrode–electrolyte phenomena of the interfaces was analysed by electrochemical impedance spectroscopy (EIS). Electrochemical impedance spectroscopy (EIS) is widely used to investigate the interfacial properties of materials and the interpretation of phenomena such as the corrosion or behaviour of coatings on metallic substrates. This technique directly relates measurements of impedance and phase angle as functions of the frequency, voltage, or current applied.

**Figure 2:**
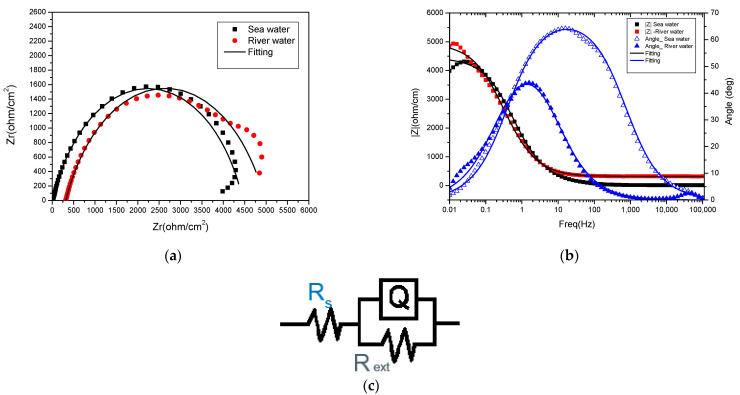
(**a**) Nyquist, (**b**) Bode impedance spectra of the reinforcing steel samples and (**c**) equivalent circuit used for fitting the data.

**Figure 3:**
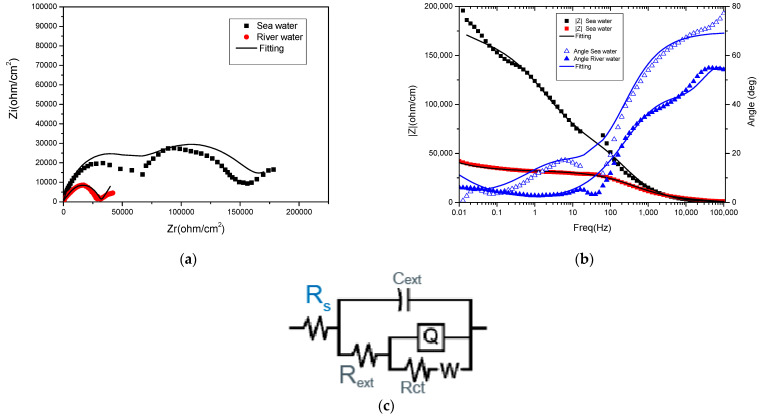
(**a**) Nyquist, (**b**) Bode impedance spectra of the solution II/Zn samples and (**c**) equivalent circuit used for fitting the data.

The Nyquist and Bode impedance spectra for the selected samples—reinforcing steel and those treated with phosphating solution II (Zn-based)—in both electrolyte solutions are presented in Figure 2 and Figure 3. Equivalent circuit element values for reinforced steel and Sol. II/Zn are presented in Table 4.

The Nyquist plot for the reinforcing steel sample (Figure 2a) shows a depressed semicircle in the high frequency range and a small inductive loop in the low frequency range. This inductive loop can be attributed to the adsorption of ions, neutral molecules in the solution, but it can also indicate the existence of intermediate reactions related to the adsorption of aggressive chlorine ions, which produce an instability of the sample surface, which is indicative of a lower corrosion resistance [25]. Also, in this case, the Bode diagram highlights the existence of a single maximum on the curve θ = f(ν) (with the phase angle as a function of the applied signal frequency), implying the existence of a single time constant, which indicates a homogeneous surface structure.

Figure 3a shows the Nyquist spectrum for the solution II/Zn sample, which displays two capacitive semicircles. These complex plane plots are obtained for the case of two adsorbed species. Also, in this case, the Bode plot (Figure 3b) highlights the existence of two maxima on the slope; this suggests the existence of two different constants. The resulting spectrum described shows two time constants, which is given by the structure of the deposited state, the porosity, or the diffusive limitations caused by the charge transfer process.

The circuit shown in Figure 2c was used for processing the EIS data obtained for the reinforcing steel samples in the river and sea water electrolytes. The impedance spectra were fitted using an equivalent circuit model (R(QR)), where Rs is the solution resistance, Q is the capacitive behaviour of the double-layer electric capacitance at the steel/solution interface and is modelled as CPE to account for actual and surface roughness, and R_ext_ is the charge transfer resistance, which is indirectly proportional to the corrosion rate (i.e., the higher the Rp, the better the steel corrosion resistance). The solution resistance (Rs) values reflect the ionic conductivity of the test medium. Higher Rs values are observed in river water (approximately 317 Ω/cm^2^) due to its lower ionic content, while lower Rs values are observed in seawater (approximately 13 Ω/cm^2^), which is consistent with its higher salinity and ionic conductivity. The charge transfer resistance (R_ext_) values for the steel samples were 4655 Ω in river water and 4447 Ω in seawater. These relatively similar R_ext_ values suggest that the initial corrosion behaviour is comparable in both environments, although slightly higher resistance was observed in river water, likely due to its lower chloride content. These results confirm that uniform corrosion predominates under the test conditions.

For the phosphate-coated steel samples, the impedance spectra were fitted using the equivalent circuit model R(C(R(Q(RW)))), as shown in Figure 3c. This circuit takes into account the capacitance (C) and resistance of the coating layer, the double-layer capacitance at the coating/substrate interface (Q), the polarisation resistance, and ionic diffusion through the porous structure of the coating layer and the corrosion products. The Warburg element (W) represents this diffusion. The inclusion of the Warburg element is justified by the controlled diffusion behaviour observed at low frequencies in the impedance spectra. The solution resistance (Rs) values obtained for the phosphate-coated samples were consistent with the expected trend, i.e., higher Rs (366 Ω·cm^2^) in river water due to lower ionic conductivity and lower Rs in seawater (175 Ω·cm^2^). Capacitance (C) values offer an insight into the dielectric properties and integrity of phosphate coatings. Lower capacitance values (around 1.68 × 10^−9^ F/cm^2^) suggest a degraded coating, especially in a more aggressive environment such as seawater, while higher capacitance values (around 4.12 × 10^−9^ F/cm^2^) indicate partial degradation of the coating. Charge transfer resistance values reflect the protective performance of phosphate coatings. In river water, a high R_ext_ of ~12,120 Ω/cm^2^ indicates effective corrosion protection provided by the coating. In contrast, the lower Rp value of ~2734 Ω/cm^2^ observed in seawater suggests a reduction in barrier properties. The R_ct_ values obtained from the equivalent circuit fitting provide additional insight into the corrosion protection effectiveness of phosphate coatings. In river water, a high R_ct_ of ~64,830 Ω indicates a good corrosion resistance at the metal/coating interface. In contrast, the lower R_ct_ of ~28,770 Ω observed in seawater suggests reduced interface protection. The Warburg impedance parameter (W) provides valuable information on the degree of ionic diffusion through the phosphate layers and any underlying corrosion products. In this study, a low value of W of ~1.64 × 10^−5^ S-s^1/2^ was observed in river water, consistent with the compact structure of the phosphate layer and minimal ion transport through the layer. In contrast, the significantly higher W value of ~3.59 × 10^−4^ S-s^1/2^ recorded in seawater indicates enhanced ionic diffusion, probably due to increased porosity or partial degradation of the layer under chloride-rich conditions.

### 3.2. Morphology and Structure Analysis of Phosphate Layers After Corrosion

A scanning electron microscope equipped with an EDAX X-ray detector was used to evaluate the morphological surface modification of the samples after corrosion. For comparison, two samples were analysed: reinforcing steel corroded in seawater and the sample phosphated with solution II in the same electrolyte.

**Figure 4:**
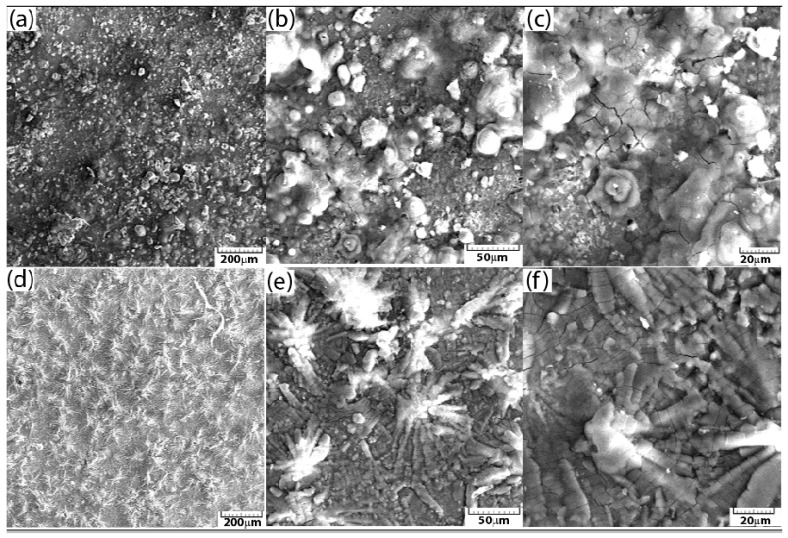
SEM images at different magnifications after the corrosion process in seawater for reinforcing steel at (**a**) 100×, (**b**) 500×, and (**c**) 1000× and for the solution II phosphated sample at (**d**) 100×, (**e**) 500×, and (**f**) 1000×.

The surface condition of the reinforcing steel sample at different image magnifications of 100×, 500×, and 1000× after the corrosion resistance test in seawater is shown via SEM microscopy in Figure 4a–c.

**Figure 5:**
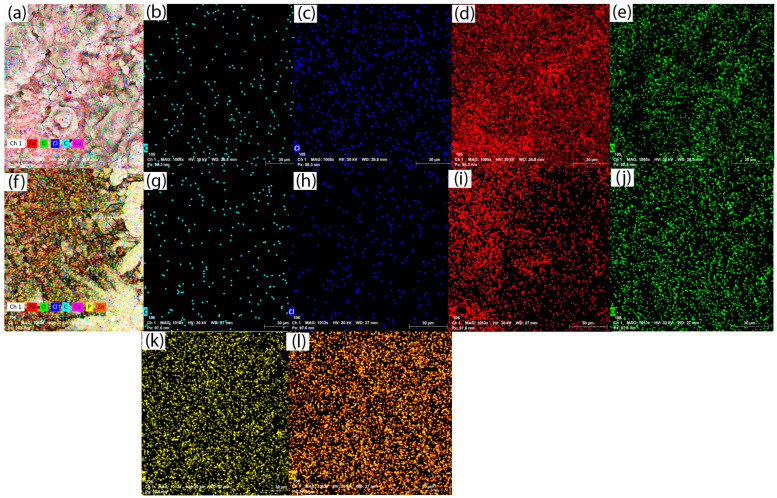
Main element distribution after the corrosion test of reinforcing steel sample: (**a**) elemental distribution; (**b**) carbon distribution; (**c**) chlorine distribution; (**d**) iron distribution; (**e**) oxygen distribution; and for the solution II phosphated sample: (**f**) element distribution; (**g**) carbon distribution; (**h**) chlorine distribution; (**i**) iron distribution; (**j**) oxygen distribution; (**k**) phosphorus distribution; (**l**) zinc distribution.

Figure 4d–f shows the SEM images of the II/Zn solution phosphated sample that showed the best corrosion resistance at different amplifications after the corrosion process in seawater. It is observed that the coating did not suffer any major damage after the corrosion test, and no corrosion marks or exfoliation were observed on the surface. The formation of micron-sized compounds on the surface during the corrosion process can also be observed in this case. The thickness of the deposited layer is ~10 μm, and that of the conversion layer is ~20–30 μm. It can be observed that the material suffered significant damage during the corrosion test, caused by the deposition of iron oxide and hydroxide corrosion compounds on its surface. From the element distribution, shown in Figure 5, the presence of chlorine and oxygen on the surface of reinforcing steel can be noted.

**Figure 6:**
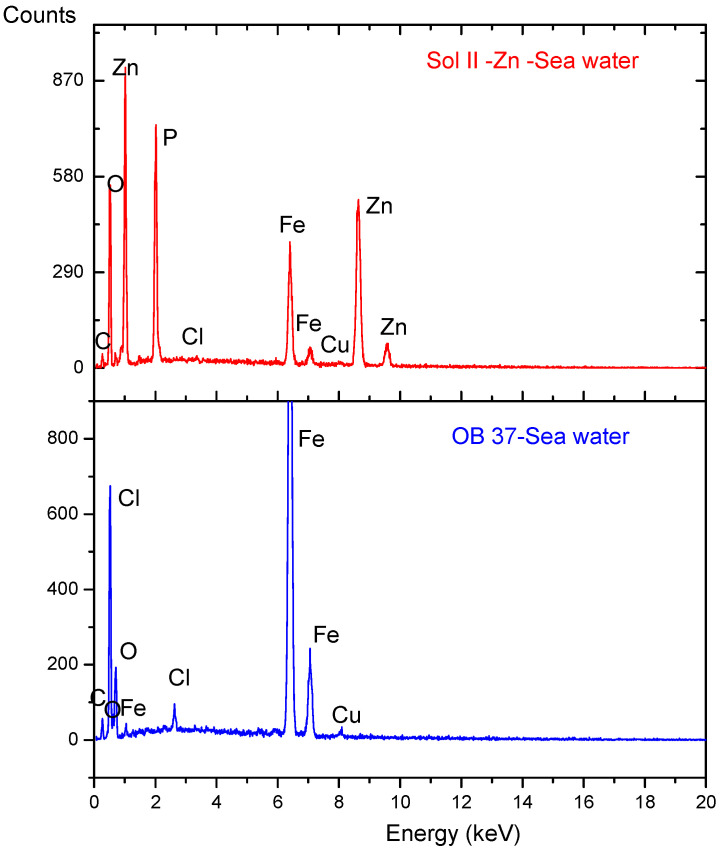
Energy spectra of reinforcing steel sample and of solution II phosphated sample after the corrosion test.

From the energy spectra in Figure 6 and from the chemical composition obtained from the corroded surface, the presence of O and chlorine in high percentages and the decrease in iron mass due to the oxidation process can be observed, as shown in Table 5.

From the element distribution shown in Figure 5f–l, the presence of chlorine, oxygen, and phosphorus on the surface of the layer can also be observed.

From the surface chemical composition of the sample phosphated with solution II, presented in Table 6, a decrease in the chlorine element percentage is observed, indicating that the sample has not been impacted as severely by Cl ions, and chloride-based corrosion compounds are present in lower percentages than those noted for the corrosion of reinforcing steel. The high percentage of carbon can be attributed to errors in the EDS detector due to surface contamination from atmospheric exposure. Additionally, no carbon agglomerations, such as those specific to surface carbonates, were observed in the distribution shown in Figure 5g.

## 4. Discussion

For reinforcing steel control sample, the cyclic diagrams obtained in the two corrosion environments showed different behaviours. The diagram in river water revealed a generalised corrosion process over the entire surface of the sample, without the occurrence of the passivation phenomenon. The cyclic diagram recorded in seawater showed a pitting corrosion character, with the current density value increasing significantly when metal oxidation processes occurred. At higher potentials, oxygen evolution can also occur at the same time as metal oxidation [17]. This can be attributed to the greater presence of Cl ions in seawater.

For the reinforcing steel sample phosphated with I/Mg phosphate solution, the layer was enriched with Mg ions. A similar behaviour was observed for the linear polarisation of the samples in the two corrosion environments, with the anodic reaction predominating, with a very high corrosion rate in the seawater electrolyte of 421 µm/year. The cyclic polarisation behaviour was similar to that of the reinforcing steel control sample, possibly due to the high reactivity of the Mg layer, leading to perforation and the electrolyte reaching the substrate [25].

For the reinforcing steel sample phosphated with II/Zn phosphating solution, no significant difference was observed between the anodic and cathodic slopes, with the metal corroding at a significantly lower rate of 0.258 µm/year. This phenomenon is the result of Zn being present in the phosphate layer, which forms ZnO, a passivating oxide, in the two corrosive media [22]. The cyclic polarisation curves recorded in both corrosion media are characteristic for a pitting corrosion alloy, with a higher current density value in seawater due to Cl ions. After the pitting occurred, the metal started to corrode.

For the reinforcing steel sample phosphated with III/Mn phosphating solution, no significant difference between the anodic and cathodic slopes was observed, with the layer corroding at a much slower rate than for the reinforcing steel control sample. From the cyclic polarization curves, a difference in corrosion behaviour was observed in both environments, with the samples exhibiting a generalized corrosion character. This phenomenon could be caused by the complex composition of the Ni, Fe, and Mn phosphate layer.

The morphological changes of the sample surfaces after corrosion were comparatively evaluated by analysing the corroded reinforcing steel control sample in seawater and the reinforcing steel sample phosphated with II/Zn solution in the same electrolyte.

For the control sample, it can be concluded that the material was severely damaged in the corrosion test by the deposition of corrosion compounds—iron oxides and hydroxides—on its surface. The distribution of elements revealed the presence of chlorine and oxygen on the surface of the reinforcing steel.

## 5. Conclusions

This study provides new comparative insights into the corrosion protection performance of zinc, manganese, and magnesium phosphate coatings applied to steel and exposed to realistic service environments. By systematically analysing how these coatings behave in freshwater and marine conditions, the study helps to fill existing knowledge gaps and provides practical guidance on how to optimise protective strategies in industrial applications. In this study, the corrosion behaviour of reinforcing steel was evaluated in two distinct aqueous environments: river water sourced from the Bahlui River and seawater collected from the Aegean Sea. Considering the corrosion process parameters obtained from the Tafel diagrams for all the phosphate samples and the reinforcing steel control sample, it can be concluded that the best corrosion resistance was recorded for the II/Zn solution phosphate samples, with the lowest corrosion rate in both electrolyte media of 0.258 µm/year in river water and 3.060 µm/year in seawater. The values obtained are much lower compared to the steel corrosion rates of around 45 µm/year.

For the reinforcing steel sample phosphated with II/Zn phosphating solution, which exhibited the best corrosion resistance, it was observed that the coating exhibited no significant degradation during the corrosion tests, with no visible signs of corrosion products or surface exfoliation. The formation of micron-sized compounds on the surface during the corrosion process can also be observed. The sample chemical composition showed a decrease in chlorine percentage, indicating that the sample was less severely impacted by Cl ions and that the chloride-based corrosion compounds were present in lower percentages than for the corroded reinforcing steel control sample.

## Figures and Tables

**Table 1:** Chemical composition of reinforcing steel used as substrate (wt.%), data from [1].

Element	C	Si	Mn	S	P	Fe
wt.%	0.23	0.07	0.75	0.045	0.045	rest

**Table 2:** The composition of the three phosphating solutions used in the experimental research, data from [1].

Solution I	Solution II	Solution III
NaOH (7 g)	NaOH (0.9 g)	NaOH (0.75 g)
MgCO_3_ (8.5 g)	NaNO_2_ (0.6 g)	NaNO_2_ (0.45 g)
NaNO_2_ (0.4 g)	Na_5_P_3_O_10_ (0.1 g)	Na_5_P_3_O_10_ (0.05 g)
H_3_PO_4_ (85%, 23 mL)	H_3_PO_4_ (22 mL)	H_3_PO_4_ (7 mL)
	HNO_3_ (11 mL)	HNO_3_ (0.4 mL)
	Zn (9 g)	Ni (0.03 g)
		Fe (0.03)
		Mn (1.5 g)

**Table 3:** Instantaneous corrosion process parameters depending on the electrolyte.

Parameter	Reinforcing Steel		Sol. I—MgCO_3_		Sol. II—Zn		Sol. III—NiFeMn	
	River	Seawater	River	Seawater	River	Seawater	River	Seawater
E_r_, mV(SCE)	−650.6	−686.6	−451.1	−514.3	−353	−386.8	−492.0	−429.9
Corrosion current density, µA/ cm^2^	3.937	3.762	10.393	23.221	0.028	0.187	3.78	3.059
Rp, kohm/cm^2^	13.25	7.78	5.52	2.64	291	154.18	9.27	18.54
Corrosion rate, µm/year	45.35	43.34	226.35	421.47	**0.258**	**3.060**	47.68	40.82
β_a_, mV/dec	129	92	365	144	180	97	193	200
−β_c_, mV/dec	737	543	363	770	102	268	205	350

**Table 4:** Equivalent circuit element values for reinforced steel and Sol. II/Zn.

Sample		R_s_ Ω·cm^2^	C_ext_ F/cm^2^	CPE			R_ext_ Ω·cm^2^	CPE	R_ct_ Ω·cm^2^	W Ss^1/2^/cm^2^
				Q Ss^n^/cm^2^	n		Q Ss^n^/cm^2^	n		
Reinforcing steel	River water	317	-	1.69 × 10^−4^	0.74	4655	-	-	-	-
	Sea water	13	-	1.09 × 10^−4^	0.77	4447	-	-	-	-
Sol. II/Zn	River water	366	1.68 × 10^−9^	-	-	12,120	1.84 × 10^−6^	0.63	64,830	1.64 × 10^−5^
	Sea water	175	4.12 × 10^−9^	-	-	2734	2.92 × 10^−7^	0.70	28,770	3.59 × 10^−4^

**Table 5:** Surface chemical composition of reinforcing steel after corrosion with seawater as electrolyte solution.

Element	At. No.	wt [%]	at [%]
Fe	26	57.77	27.26
O	8	33.24	54.85
C	6	7.708	16.94
Cl	17	1.262	0.93
Total		100	100

**Table 6:** Surface chemical composition of reinforcing steel phosphated with solution II after corrosion with seawater as the electrolyte solution.

Element	At. No.	wt [%]	at [%]	EDS Error [%]
O	8	34.75	55.72	6.10
Zn	30	32.95	12.93	0.95
P	15	13.45	11.14	0.63
Fe	26	11.88	5.29	0.46
C	6	6.88	14.71	3.18
Cl	17	0.06	0.04	0.06

## Data Availability

The original contributions presented in this study are included in the article. Further inquiries can be directed to the corresponding authors.
